# Ginsenoside Rb3 provides protective effects against cisplatin‐induced nephrotoxicity via regulation of AMPK‐/mTOR‐mediated autophagy and inhibition of apoptosis in vitro and in vivo

**DOI:** 10.1111/cpr.12627

**Published:** 2019-05-16

**Authors:** Jing‐jing Xing, Jin‐gang Hou, Zhi‐na Ma, Zi Wang, Shen Ren, Ying‐ping Wang, Wen‐cong Liu, Chen Chen, Wei Li

**Affiliations:** ^1^ College of Chinese Medicinal Materials Jilin Agricultural University Changchun China; ^2^ National & Local Joint Engineering Research Center for Ginseng Breeding and Development Changchun China; ^3^ Intelligent Synthetic Biology Center Daejeon Korea; ^4^ School of Biomedical Sciences University of Queensland Brisbane Queensland Australia

**Keywords:** AMPK/mTOR, autophagy, cisplatin, Ginsenoside Rb3, HEK293 cells, nephrotoxicity

## Abstract

**Objectives:**

Based on previous reports that ginsenosides have been shown to exert better preventive effects on cisplatin‐induced kidney injury, the present work aims to evaluate the protective effects of ginsenoside Rb3 (G‐Rb3) on cisplatin‐induced renal damage and underlying mechanisms in vivo and in vitro*.*

**Materials and methods:**

The protective effect of G‐Rb3 on cisplatin‐induced acute renal failure in ICR mouse model and HEK293 cell model was investigated, and the underlying possible mechanisms were also explored. For animal experiment, renal function, kidney histology, inflammation, oxidative stress, relative protein molecules involved in apoptosis and autophagy signalling pathways were assessed. In addition, rapamycin (a specific inhibitor of mTOR), compound C (a specific inhibitor of AMPK) and acetylcysteine (NAC, a specific ROS scavenger) were employed to testify the effects of AMPK/mTOR signal pathway on the protective effects of G‐Rb3 in HEK293 cells.

**Results:**

Pre‐treatment with G‐Rb3 at doses of 10 and 20 mg/kg for ten days significantly reversed the increases in serum creatinine (CRE), blood urea nitrogen (BUN) and malondialdehyde (MDA), and decrease in glutathione (GSH) content and superoxide dismutase (SOD) activity. Histopathological examination further revealed that G‐Rb3 inhibited cisplatin‐induced nephrotoxicity. G‐Rb3 diminished cisplatin‐induced increase in protein expression levels of p62, Atg3, Atg5 and Atg7, and decrease in protein expression level of p‐mTOR and the ratio of LC3‐I/LC3‐II, indicating that G‐Rb3 suppressed cisplatin‐induced activation of autophagy. Inhibition of autophagy induced inactivation of apoptosis, which suggested that autophagy played an adverse effect on cisplatin‐evoked renal damage. Further, we found that G‐Rb3 might potentially modulate the expressions of AMPK‐related signal pathways.

**Conclusions:**

These findings clearly suggested that G‐Rb3‐mediated alleviation of cisplatin‐induced nephrotoxicity was in part due to regulation of AMPK‐/mTOR‐mediated autophagy and inhibition of apoptosis in vitro and in vivo.

## 1 INTRODUCTION

Cisplatin is one of the most widely used chemotherapeutic agents in clinic against various cancers.^1^ However, its clinical application was greatly limited due to its cumulative renal toxicity.^2^ Although various approaches have been suggested to overcome such toxicity, to date no effective medical treatment has been established. Hence, it is urgent to identify a new treatment to protect patients under cisplatin‐based chemotherapy from renal toxicity.

Oxidative stress is an imbalance in the redox reaction, leading to the generation of ROS in the pathophysiology of cisplatin‐evoked kidney injury.^3^ A growing body of evidence illustrated that excessive accumulation ROS and decrease in antioxidants such as GSH and SOD contributed to kidney pathological injuries including oxidative stress, mitochondrial damage and apoptosis in cisplatin‐induced renal injury in animal model.^4^ Simultaneously, cisplatin induced DNA damage in the kidney, which was related to the process of apoptosis. Apoptosis is an evolutionarily conserved process.^5^ The apoptotic cascade was triggered by cisplatin through the intrinsic pathway, which involved the mitochondrial failure through accumulating ROS.^6^ Autophagy plays an important role in the maintenance of cellular homeostasis, involved in fundamental biological activities.^7^ The formation of autophagosome including autophagy‐related genes and the conversion of LC3‐I to LC3‐II through photolytic cleavage and lipidation are considered as hallmarks of mammalian autophagy.^8^ Previous studies illustrated that activation of autophagy stimulated ROS formation and that cisplatin exposure exerted a synergistic effect on ROS accumulation.^9^ AMP‐activated protein kinase (AMPK), the energy sensor in many cells, is activated by autophagy‐related cell stress and promotes metabolic reprogramming. Here, mTOR kinase plays a critical role in inhibition of autophagy induction.^10^ Once energy homeostasis is disturbed, activated AMPK may suppress mTOR activity to relieve the inhibition of autophagy.^11^ Several other signalling pathways such as Akt/mTOR have been testified to regulate autophagy‐mediated dysfunction of diabetic tubular damage.^12^ More recent studies confirmed that autophagy and apoptosis played pivotal roles in nephrotoxicity development. Therefore, this study focused on the autophagy and apoptosis in order to search for the novel therapeutic options.

The pathogenesis and underlying mechanism of AKI remain poorly understood. Several compounds with free radical scavenging and antioxidant capabilities have been tested to attenuate the cytotoxic effect of cisplatin. Recently, traditional Chinese herbal medications (TCHMs) have frequently shown specific beneficial effects on cisplatin‐induced nephrotoxicity and ischaemia‐reperfusion cardiomyocytes.^13^, ^14^ The ginseng leaf is rich in ginsenosides and has potential application for its antioxidant capacity.^15^ Previous studies also suggested that Rb2 alleviated hepatic lipid accumulation by restoring autophagy via the induction of sirt1 and activation of AMPK, and resulted in improved non‐alcoholic fatty liver disease (NAFLD) and glucose tolerance.^16^ Supplementation of saponins from leaves of *Panax quinquefolius* mitigated cisplatin‐evoked nephrotoxicity by suppressing ROS‐mediated activation of MAPK and NF‐κB signal pathways.^14^ Ginsenoside Rb3 (G‐Rb3) is a major and representative one of protopanaxadiol triterpenoid saponin.^17^ However, the potential protective effects of G‐Rb3 on cisplatin‐induced AKI were poorly investigated till now.

Ginsenoside, as the major active constituents in ginseng, possesses many therapeutic effects including anti‐diabetes, anti‐tumour, antioxidation and anti‐apoptosis activities.^18^ In the present work, the protective effects of G‐Rb3 against cisplatin‐induced renal toxicity were reported for the first time in a mouse model and in HEK293 renal cells. The results from the work clearly suggested that the beneficial effects of G‐Rb3 on renal damage might closely relate to the inhibition of ROS‐induced apoptosis and autophagy through AMPK/mTOR signalling pathways.

## 2 MATERIALS AND METHODS

### 2.1 Chemicals and reagents

G‐Rb3 (purity ≥ 98.5%, HPLC method) was isolated and purified from the leaves of *Panax quinquefolium* (American ginseng). Cisplatin was purchased from Sigma Chemicals with purity more than 99%. Compound C, rapamycin (Ram) and acetylcysteine (NAC) also were purchased from MedChemExpress Biotech and stored at −80°C in darkness. The commercial assay kits for determining reduced glutathione (GSH), superoxide dismutase (SOD), malondialdehyde (MDA), blood urea nitrogen (BUN) and creatinine (CRE) were bought from Nanjing Jiancheng Biological Research Institute. Haematoxylin and eosin (H&E) dying kit and Hoechst 33258 staining kit were obtained from Beyotime Co, Ltd. The immunohistochemically assay kits together with SABC‐DyLight488 immunofluorescence staining kits were obtained from BOSTER Biological Technology Co, Ltd. The primary rabbit monoclonal antibodies including anti‐LC3, anti‐BNIP3, anti‐β‐actin, anti‐GAPDH, anti‐Atg3, anti‐Atg5, anti‐Atg7 and anti‐p62 were all provided by BOSTER Biological Technology Co, Ltd. The rabbit anti‐AMPK, rabbit anti‐mTOR, rabbit anti‐Bax, Bcl‐2, Bad, caspase 3 and caspase 9 were acquired from Cell Signaling Technology. TUNEL commercial kit was purchased from Roche Applied Science. All other reagents and chemicals, unless indicated, were obtained from Beijing Chemical Factory.

### 2.2 Animal and experiments design

Male adult ICR mice weighing 22 ~ 25 g, SPF grade, were provided by Changchun YISI Experimental Animal Holding with a Certificate of Quality No. of SCXK (JI)‐2016‐0003 and raised at temperature of 23.0 ± 2.0°C on 12 hours light‐dark cycle with free access to food and water. All experimental animals’ processing project was strictly performed according to the Guide for the Care and Use of Laboratory Animals (2016). The mice were allowed to adapt the environment for 7 days. All mice were fed with a standard diet and tap water. All animals’ protocols were in accordance with the Ethical Committee for Laboratory Animals of Jilin Agricultural University. The mice were randomly divided into four groups (n = 8): normal group, cisplatin group and G‐Rb3 groups (10 and 20 mg/kg), respectively. G‐Rb3 powder was dissolved in 0.05% carboxymethylcellulose sodium (CMC‐Na). G‐Rb3 was orally administrated to all groups except for normal groups for 10 continuous days. In the meantime, the mice in normal and cisplatin group were administered with 0.05% CMC‐Na by oral administration once daily. On the 7th day, a single dose of cisplatin (25 mg/kg, dissolved in water) was intraperitoneally injected to mice in cisplatin group and G‐Rb3 groups to induce acute renal damage after 1 hour last administration. On the 10th day, mice were fasted overnight. All groups were euthanized 72 hours after exposure to cisplatin. Serum samples were collected by the retrobulbar vessels immediately and placed at room temperature for 45 minutes and separated by centrifugation for 10 minutes at 3000 *g* under 4°C for analysis of biochemical parameters. Then, kidney tissues in all groups were immediately dissected out, washed with cold saline, blotted on a filter paper and measured for weights. The left kidney was immersed in 10% neutral buffered formalin for tissue sections. The right kidney was promptly frozen in liquid nitrogen and stored at −80°C for further kidney homogenate for subsequent measurement of kidney MDA and GSH and SOD levels, and Western blot analysis.

### 2.3 Cell culture and treatment

In order to evaluate the protective effect against renal damage in vitro, HEK293 cell line (human embryonic kidney epithelial cells) obtained from ATCC was employed in the present experiment. The cells were cultured in DMEM containing 10% FBS at 37°C in a 5% CO2 atmosphere at 37°C. At day 3 of culture, cells were seeded on 96‐well culture plate and the cells grow to approximately 70%~80% confluence in complete medium containing 10% FBS for 24 hours. Cultures were supplemented with G‐Rb3 with different concentration of 0.25, 0.5, 1.0 and 2.0 μmol/L for 24 hours. Next, cells were then treated with 20 μmol/L cisplatin (dissolved with serum‐free medium) after washing twice with serum‐free medium for 24 hours. Cell viability was measured by MTT assay according to the manufacturer with slight modifications.^19^ After exposure to cisplatin with or without G‐Rb3 for 24 hours, the dark‐blue formazan crystals formed in the cells were dissolved in DMSO. Absorbance was recorded at 490 nm by using a microplate reader (Nano, Germany). Cell viability was expressed as a percentage of the absorbance of wells treated with cisplatin alone. The HEK293 renal cells were specifically seeded into six groups: normal group, G‐Rb3 group and Ram/compound C/NAC group (200 nmol/L, 20 μmol/L and 1 mmol/L, respectively), Cisplatin group (1 μmol) , Cisplatin + Ram/compound C/NAC group (200 nmol/L, 20 μmol/L and 1 mmol/L, respectively), Cisplatin + Rb3 + Ram/compound C/NAC group (200 nmol/L, 20 μmol/L and 1 mmol/L, respectively). Various inhibitors were given to the second group at different concentrations, and G‐Rb3 powder was dissolved in DMEM and was given to the second and sixth groups for 24 hours. In the meantime, the normal and cisplatin groups were administered with cells at a concentration of cisplatin (20 μmol/L, dissolved in water). After treatment, 20 μL MTT solution (5 mg/mL) was added to the wells and incubated at 37°C for 4 hours. Then, 150 μL DMSO was added to each well, and the plates were agitated for 5 minutes. Finally, the absorbance was measured at 490 nm using a microplate reader (Nano, Germany). The intensity of the colour produced was proportional to the number of living HEK293 cells.

### 2.4 H&E staining

Kidney samples were fixed in 10% formalin, embedded in paraffin solution and then sectioned in 5 µmol/L. Then, the sections were stained with H&E dye kits for general histology and examined under a digital camera and photographed (Leica TCS SP8). Then, randomly chosen fields (×100 and ×400) of slides were evaluated for each specimen, and an average score was calculated. Histopathological changes were blindly scored by a pathologist as previously described^20^ on a 5‐point scale: 0 = no damage, 1 = 10% of junction injured, 2 = 10~25%, 3 = 25~50%, 4 = 50~75 and 5 = more than 75%.

### 2.5 Estimation of lipid peroxidation

Kidney tissues were removed from −80°C and homogenized in ice‐cold phosphate buffer (pH 7.4), and the homogenates obtained were then used to determine enzyme activity. The levels of GSH and MDA, and SOD were analysed using commercial reagent kits according to the manufacturer's protocols. Samples containing lipid peroxidation were mixed with thiobarbituric acid (TBA) to form red mixture. The tissues were homogenized with 0.9% NaCl and centrifuged (3000 *g*, 30 minutes at 4°C). The levels of GSH, SOD and MDA were measured with centrifuged supernatants using the corresponding commercial kits as described previously.

### 2.6 Biochemical parameters

Serum markers of kidney damage, creatinine (CRE) and blood urea nitrogen (BUN) were examined by commercial kits (Jiancheng).

### 2.7 Immunohistochemistry and TUNEL staining

Immunohistochemical analysis was performed as previously described.^21^ The tissue samples were deparaffinized and rehydrated, and then treated with citrate buffer solutions (0.01 mol/L, pH 6.0) for 20 minutes. After washing with PBS (0.01 mol/L, pH 7.4) for three times, these sections were incubated with 1% bovine serum albumin (BSA) for 10 minutes to block on specific binding at room temperature. Then, the renal sections were incubated at 4°C overnight. The slides were immunostained with primary antibodies against Bax (1:200) and Bcl‐2 (1:200), respectively. The slides were then stained with TRITC‐ or FITC‐conjugated secondary antibodies. Slides were viewed with light microscope (Leica, DN750, Soles, and Germany).

We detected apoptosis cells by employing terminal deoxynucleotidyl transferase‐mediated dUTP nick end‐labelling (TUNEL) staining according to the manufacturer's protocol (Roche Company). Briefly, the kidney sections were fixed with proteinase K (20 mg/mL) in distilled water for 10 minutes. The fixed kidney tissues were incubated in methanol including 3% hydrogen peroxide (H_2_O_2_) for 20 minutes to block endogenous peroxidase. The slides were treated with equilibration buffer solution and terminal deoxynucleotidyl transferase, and then, anti‐digoxigenin‐peroxidase conjugate was added to fixed kidney sections. Peroxidase activity in each kidney section was stained by DAB, and haematoxylin was used to counterstain the sections.

### 2.8 Immunofluorescence staining and Hoechst 33258

Kidney sections at 5 μm thickness were deparaffinized and rehydrated, and then treated with citrate buffer solution (0.01 mol/L, pH 6.0) for 20 minutes. After washing with PBS for three times, the sections were incubated with 1% BSA for 10 minutes to block non‐specific binding at room temperature. Then, the kidney sections were incubated at 4°C overnight with primary antibodies including rabbit anti‐BNIP3 antibody (1:200 dilution) and rabbit anti‐LC3 antibody (1:200 dilution) followed by secondary antibody for 30 minutes. After washing with PBS for three times, the slices were incubated with DyLight after 12 hours at 37°C. Then, nuclear structures were exposed to 4, 6 diamidino‐2‐phenylindole (DAPI) staining. Images were captured by a Leica microscope (Leica DFC 450 C).

Hoechst 33258 staining was performed as previously described with some modifications.^22^ Briefly, renal tissues with 5 μm thickness were dissected out and fixed in 10% neutral buffered formalin solution. We randomly chose three samples from each group. Then, these samples were stained by Hoechst 33258 (10 g/mL). After being washed with PBS for three times, stained nuclei were visualized under UV excitation and photographed under a fluorescent microscope (Olympus BX‐60).

### 2.9 ROS staining

The relative levels of intracellular ROS were determined by a fluorometric assay (DCF‐DA assay, Wanlei Biotechnology) according to the manufactures’ protocols. HEK293 cells were seeded in 6‐well microplates, and treated HEK293 cells were incubated with 1.0 μmol/L DCFH‐DA at 37°C for 12 hours. Then, the medium was removed and cells were washed by PBS twice prior to imaging. Relative DCF fluorescence intensity of treated cells was expressed as a percentage of cisplatin‐induced group (Leica TCS SP8).

### 2.10 Western Blot

To elucidate the nephroprotective mechanism of G‐Rb3 on kidney injury induced by cisplatin, modulation of protein expression was studied. The collected kidneys were lysed in ice‐cold RIPA buffer and were supplemented with protease inhibitor and phosphatase inhibitor. An equal amount of protein in each sample was subjected to sodium dodecyl sulphate‐polyacrylamide gel electrophoresis gels and then transferred to polyvinylidene fluoride (PVDF) membranes. After blocking with 5% BSA for 2 hours and then, membranes were incubated with the primary antibodies against mTOR (1:500), p‐mTOR (1:1000), p62 (1:500), LC3‐II (1:1000), mTOR (1:1000), p‐mTOR (1:1000), AMPK (1:1000), p‐AMPK (1:1000), Atg3 (1:1000), Atg5 (1:1000), Atg7 (1:1000) and BNIP3 (1:1000) overnight at 4°C. The level of GAPDH (1:1000) was assessed as a loading control. After washing with TBST for three times, the membranes were incubated for 2 hours with the secondary antibodies. The protein bands were measured using Image plus 6.0 software (Media Cybernetics).

### 2.11 Statistical analysis

All data referenced were expressed as the mean ± SD and analysed with SPSS 19.0 (SPSS). Differences among experimental groups were conducted by one‐way analysis of variance (ANOVA). Statistical significance was defined as *P* < 0.05 or *P* < 0.01.

## 3 RESULTS

### 3.1 G‐Rb3 protects kidney against cisplatin‐induced renal damage

**Figure 1:**
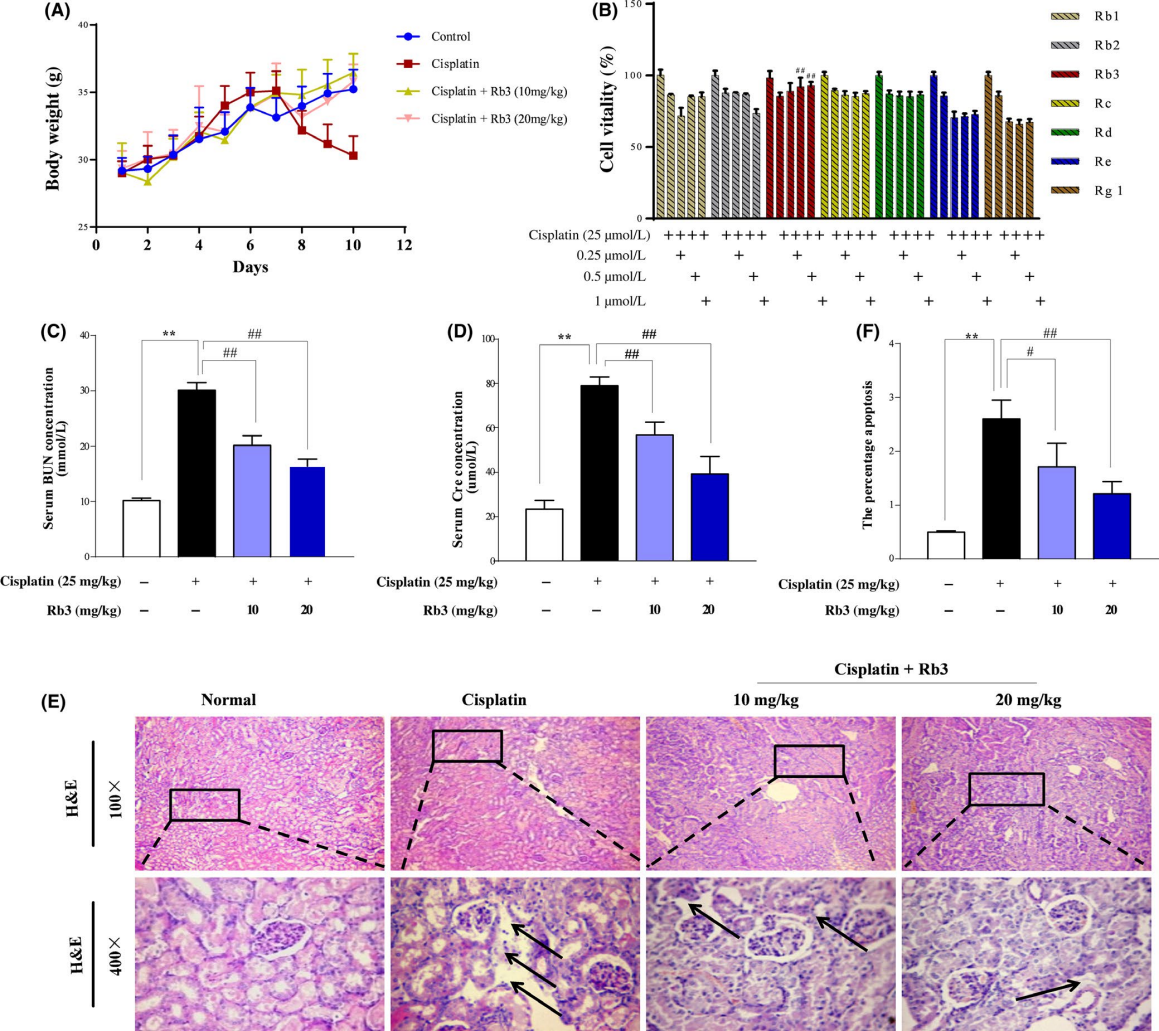
G‐Rb3 protects against cisplatin‐induced AKI. Experiment of renoprotective effect of G‐Rb3 on mice was summarized. The body weights of mice in different groups were measured (A). G‐Rb3 inhibited the decrease of vitality in HEK293 cell exposure to cisplatin. HEK293 cells were pre‐treated with Rb1, Rb2, Rb3, Rc, Rd, Re and Rg1 for 24 h and then exposed to 20 µmol/L cisplatin for another 24 h (B). MTT assay was used to detect cell viability. Effects of G‐Rb3 on the levels of blood urea nitrogen (BUN) and serum creatinine (CRE) (C‐D). Histological examinations of morphological changes were observed in kidney tissues (E), and kidney tissues were stained with haematoxylin and eosin (H&E) (100×, 400×) (F). All data are expressed as mean ± SD *P < 0.05 or **P < 0.01 comparing with normal group. ^#^ *P* < 0.05 or ^##^ *P* < 0.01 comparing with cisplatin group

**Figure 2:**
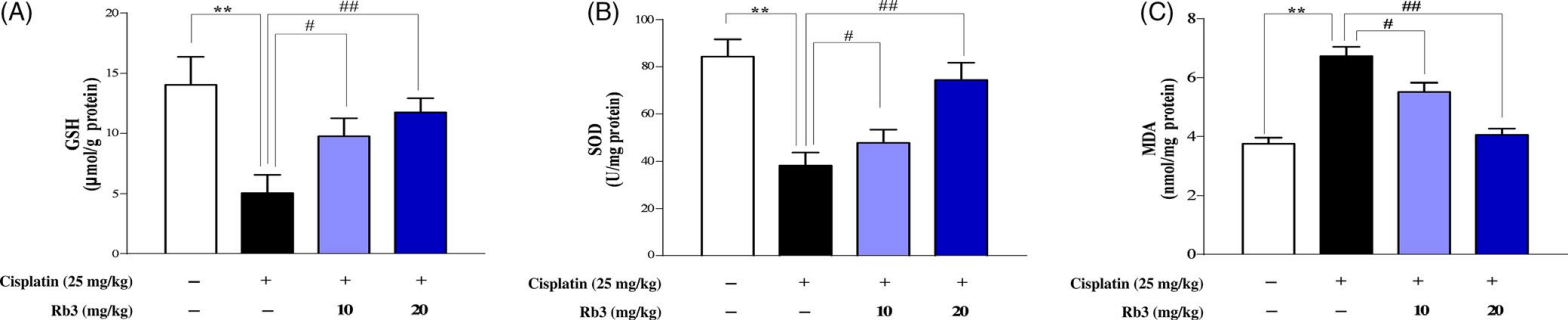
G‐Rb3 inhibits oxidative stress after cisplatin treatment. Effects of G‐Rb3 treatment on the renal levels of GSH, SOD and MDA in cisplatin‐induced AKI (A‐C). All data are expressed as mean ± SD *P < 0.05 or **P < 0.01 comparing with normal group. ^#^ *P* < 0.05 or ^##^ *P* < 0.01 comparing with cisplatin group

As shown in Figure 1A, obvious body weights loss was caused after single injection of cisplatin (25 mg/kg). However, G‐Rb3 treatment prevented these changes significantly at doses of 10 and 20 mg/kg (*P* < 0.05 or *P* < 0.01). Mice in cisplatin exposure group developed severe AKI as determined by elevated serum levels of BUN and CRE (Figure 2C,D). Treatment with G‐Rb3 (10 or 20 mg/kg) for 10 continuous days significantly attenuated cisplatin‐induced increase in serum BUN and CRE levels. Histologically, the renal tissues in cisplatin group presented significantly more necrotic regions, congestions and inflammatory cell infiltrations than that in normal group, whereas renal damage was largely diminished in mice pre‐treated with G‐Rb3 at a dose of 10 or 20 mg/kg (Figure 2E,F). Together, these results clearly confirmed a protective effect of G‐Rb3 against cisplatin‐induced renal damage in mice.

### 3.2 G‐Rb3 inhibits oxidative stress induced by cisplatin treatment

The ROS in cells played an important role in regulating physiologic tubular functions. When kidneys were exposed to cisplatin, intracellular ROS increased significantly.^23^ Cisplatin decreased the level of antioxidants such as GSH (Figure 1A) and SOD (Figure 1B), while increased the production of MDA (Figure 1C). Conversely, G‐Rb3 (10 or 20 mg/kg) reversed the decrease in renal SOD and GSH, and the overproduction of MDA.

### 3.3 G‐Rb3 reduces AKI‐induced proximal tubular apoptosis in the kidney

**Figure 3:**
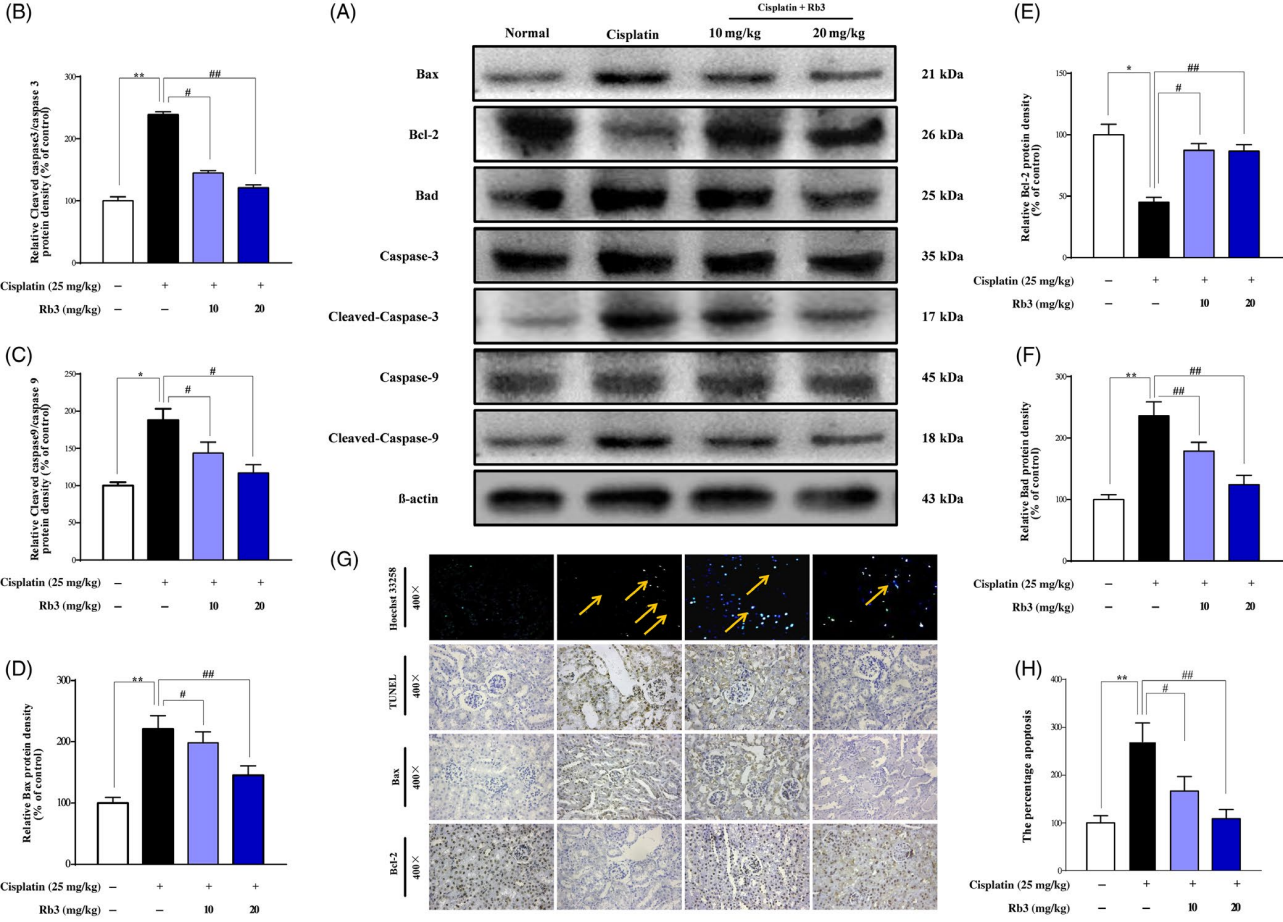
G‐Rb3 reduces tubular cell apoptosis in the kidneys with cisplatin‐induced AKI. The intensity of Bax, Bcl‐2, Bad, caspase 3, cleaved caspase 3, caspase 9 and cleaved caspase 9 was standardized to that of β‐actin (A); Quantitative analysis of scanning densitometry for cleaved caspase 3 (B), cleaved caspase 9 (C), Bax (D), Bcl‐2 (E) and Bad (F). Analysis of apoptosis in kidney tissues was evaluated by using Hoechst 33258 and TUNEL staining. The expression levels of Bax and Bcl‐2 were determined by immunohistochemical analysis (G). The degree of damage was accessed by necrosis scores (H). Kidney necrosis in mice was marked by yellow arrow. All data are expressed as mean ± SD *P < 0.05 or **P < 0.01 comparing with normal group. ^#^ *P* < 0.05 or ^##^ *P* < 0.01 comparing with cisplatin group

Tubular cell apoptosis plays an essential pathogenic role for cisplatin‐induced AKI.^1^ In this study, cell apoptosis was examined by TUNEL staining and immune staining of Bax and Bcl‐2 in kidney. As shown in Figure 3G, very few apoptotic cells were detected in the kidney sections from normal group and G‐Rb3 treatment group. However, mice injected with cisplatin alone showed significantly increase in TUNEL‐positive staining cells and overexpression of Bax protein, and decreased level of Bcl‐2 protein (Figure 3G). G‐Rb3 might protect kidney cells from apoptosis caused by cisplatin. Accordingly, the results from Western blot analysis showed that pre‐treatment with G‐Rb3 at dosages of 10 and 20 mg/kg significantly decreased the protein expression levels of Bax, Bad, caspase 3 and caspase 9, while increased the protein expression level of Bcl‐2 (Figure 3A). These results clearly suggest that G‐Rb3 pre‐treatment mitigated cisplatin‐induced tubular cell apoptosis.

### 3.4 G‐Rb3 inhibits autophagy in proximal tubules in cisplatin‐induced AKI mice

**Figure 4:**
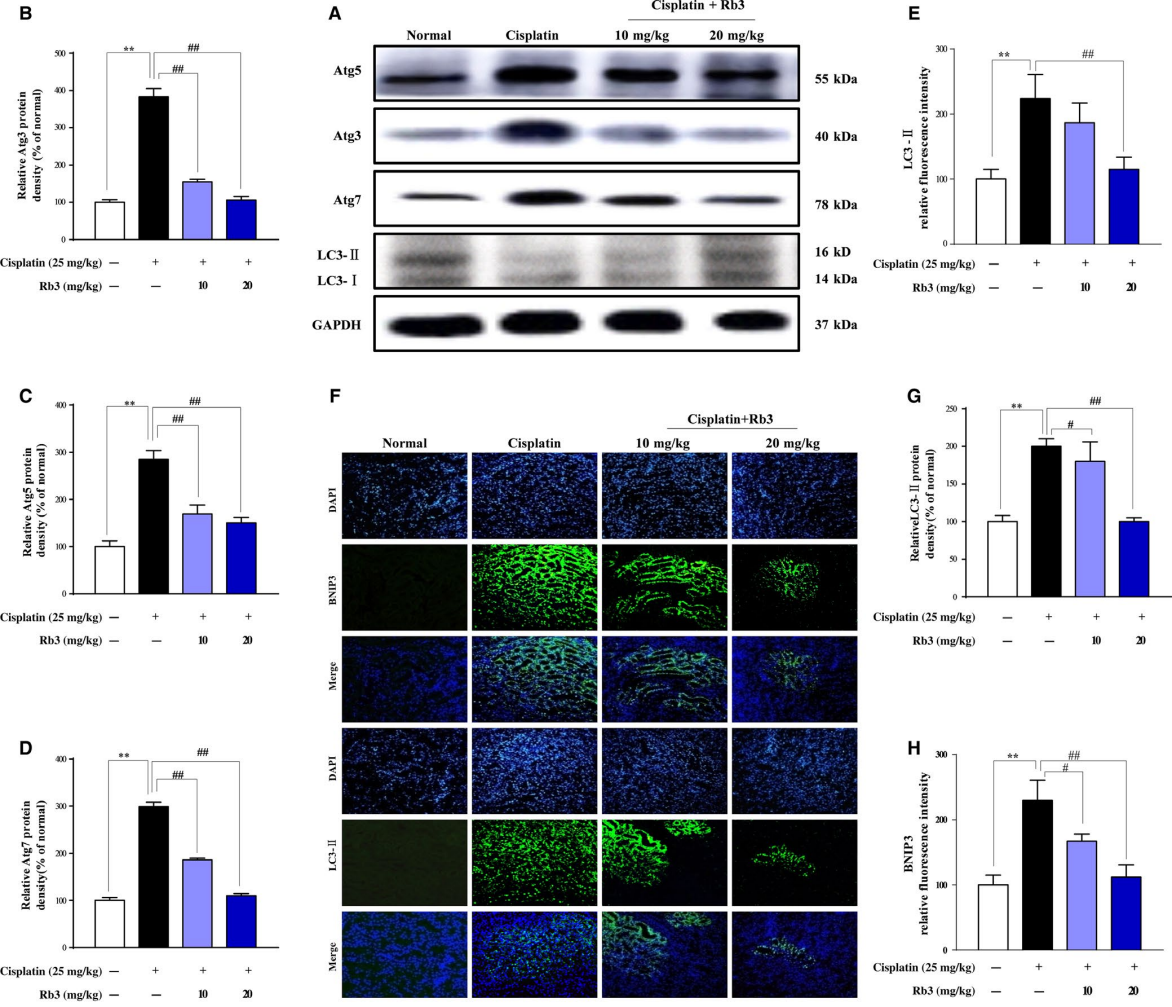
Treatment with G‐Rb3 inhibits autophagy in proximal tubules during cisplatin‐induced AKI in mice. Mice were pre‐treated with cisplatin with or without G‐Rb3 (10, 20 mg/kg). Whole tissue lysate of the kidney was collected for Western blot analysis of Atg3, Atg5, Atg7, LC3 and GAPDH. (A) The LC3, Atg3, Atg5, Atg7 and GAPDH signals in immunoblots were analysed by densitometry to calculate the ratio to be expressed as mean ± SD (B, C, D and E). *P < 0.05 vs normal group. **P < 0.01 vs cisplatin group. The expression level of LC3‐II (Green) and BNIP3 (Green) in tissue section isolated from different groups was assessed by immunofluorescence (F). Column chart (G and H) shows relative fluorescence intensity. Representative immunofluorescence images were taken at 400. 4, 6‐Diamidino‐2‐phenylindole (DAPI) (blue) was used as nuclear counterstain. All data are expressed as mean ± SD *P < 0.05 or **P < 0.01 comparing with normal group. **^#^** *P* < 0.05 or ^##^ *P* < 0.01 comparing with cisplatin group

Increased autophagosome formation is attributed to the induction of autophagy.^24^ In order to further elucidate whether autophagy played an adverse effect on AKI in mice, biochemical hallmarks of autophagy were analysed in present experiments. As shown in Figure 4, the accumulation of LC3‐I and LC3‐II was measured by immunoblots after cisplatin treatment, showing an evaluation of LC3‐I/LC3‐II ratio (Figure 4D). Consistent with the result of LC3‐II (Figure 4A), cisplatin exposure significantly elevated the protein expression levels of upstream protein molecules including Atg3, Atg5 and Atg7 (Figure 4A). Increase in these autophagy‐related proteins was decreased by G‐Rb3 treatment. Additionally, the protein expression level of BNIP3, an important morphologically factor in the formation of autophagosome in kidneys, was elevated in cisplatin group, with mass dot‐like staining in renal tubular cells located in renal cortex and outer medulla (Figure 4F,H), but reduced by G‐Rb3 treatment. These results confirmed that G‐Rb3 negatively regulated autophagy in vivo.

### 3.5 G‐Rb3 inhibits autophagy through AMPK‐/mTOR‐dependent signalling pathways

**Figure 5:**
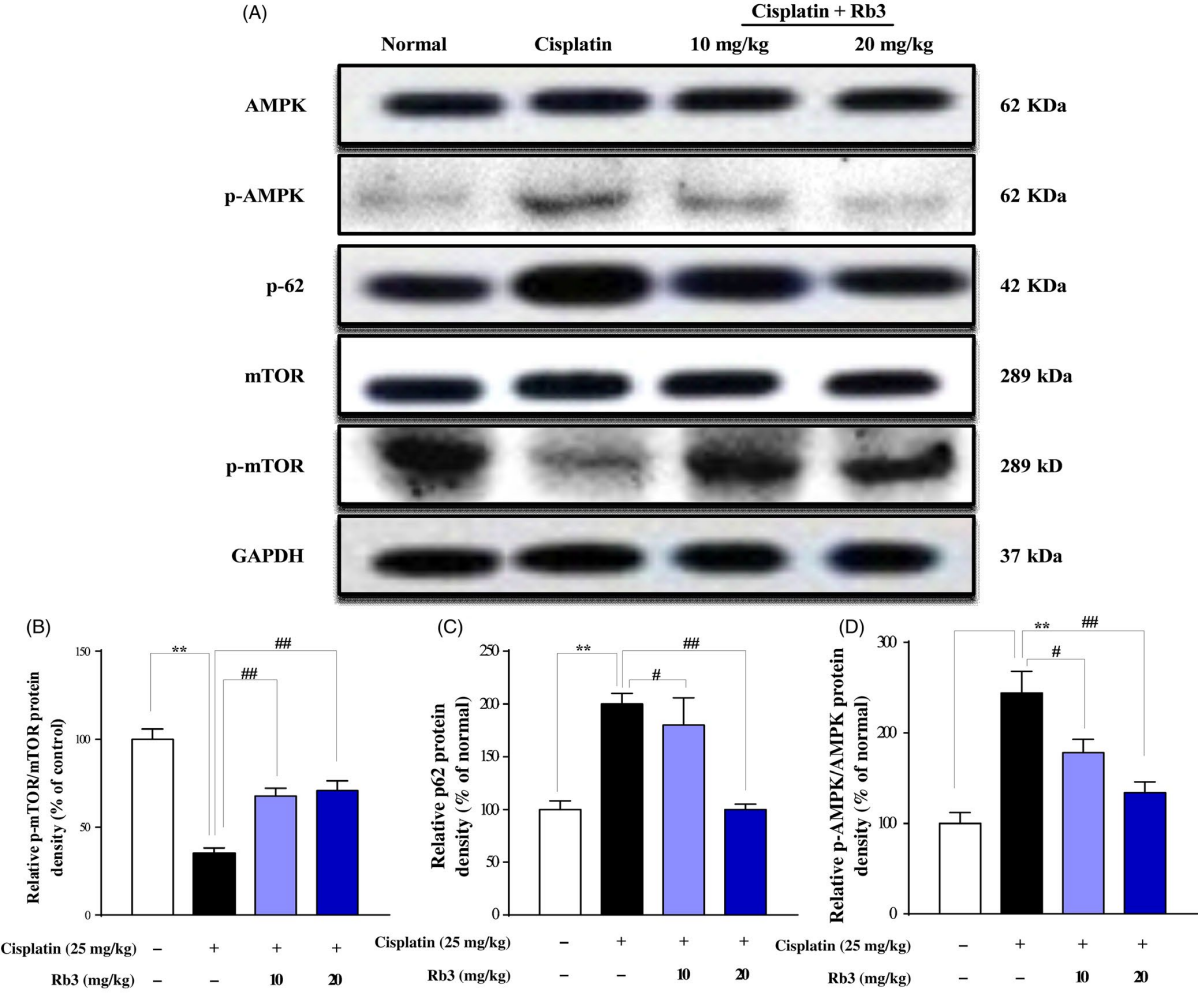
G‐Rb3 inhibits autophagy through AMPK‐/mTOR‐dependent signalling pathways in vivo. Mice pre‐treated with Rb3 or CMC‐Na for 7 d, given with or without cisplatin at day 7. Representative Western blots depicting total and phosphorylated AMPK, total and phosphorylated mTOR and p62 (A). Quantitative analyses of the p‐mTOR/mTOR, p62 and p‐AMPK/AMPK expression ratios are shown (B, C and D). All data are expressed as mean ± SD *P < 0.05 or **P < 0.01 comparing with normal group. ^#^ *P* < 0.05 or ^##^ *P* < 0.01 comparing with cisplatin group

**Figure 6:**
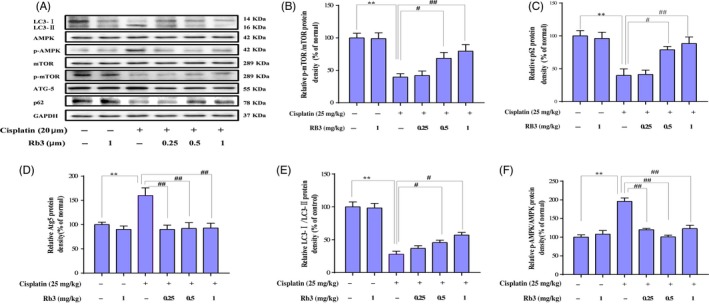
G‐Rb3 inhibits autophagy through inhibiting of AMPK/mTOR signalling pathway in cultured HEK293 cells. HEK293 cells were pre‐treated with G‐Rb3 in different concentration from 0.24 to 2.0 µm for 24 h and subjected to MTT analysis to detect cell vitality. HEK293 cells were exposed to cisplatin with or without G‐Rb3 (0.25 µm) for 24 h and subjected to Western blot analysis of AMPK, p‐AMPK, mTOR, p‐mTOR, Atg5, p62 and LC3 I/II (A‐F). All data are expressed as mean ± SD *P < 0.05 or **P < 0.05 comparing with normal group. ^#^ *P* < 0.01 or ^##^ *P* < 0.01 comparing with cisplatin group

To further determine whether the AMPK/mTOR pathway regulated cisplatin‐induced autophagy, the levels of p‐AMPK, AMPK, p‐mTOR and mTOR were analysed by Western blot in AKI mice (Figure 5). The abundance of phosphorylated AMPK (p‐AMPK) was increased slightly in cisplatin‐treated mice (25 mg/kg) and HEK293 cells (20 µmol/L), whereas pre‐treatment of G‐Rb3 in mice (10, 20 mg/kg) and cells (0.25, 0.5, 1.0 µmol/L) decreased p‐AMPK. Additionally, a key regulator of autophagy is mTOR, a nutrient‐sensing protein that inhibits autophagy. Western blot assay in vivo (Figure 5) and in vitro (Figure 6) showed cisplatin exposure decreased expression level of mTOR, while pre‐treatment with G‐Rb3 increased it, suggesting the inhibition of autophagy signalling by G‐Rb3. Moreover, LC3‐I was accumulated in kidney tissues in mice (Figure 4A) and cells (Figure 6) pre‐treated with G‐Rb3 comparing to mice and cultured cells treated with cisplatin alone.

### 3.6 Inhibition of ROS downregulates cisplatin‐evoked apoptosis in cultured HEK293 cells

**Figure 7:**
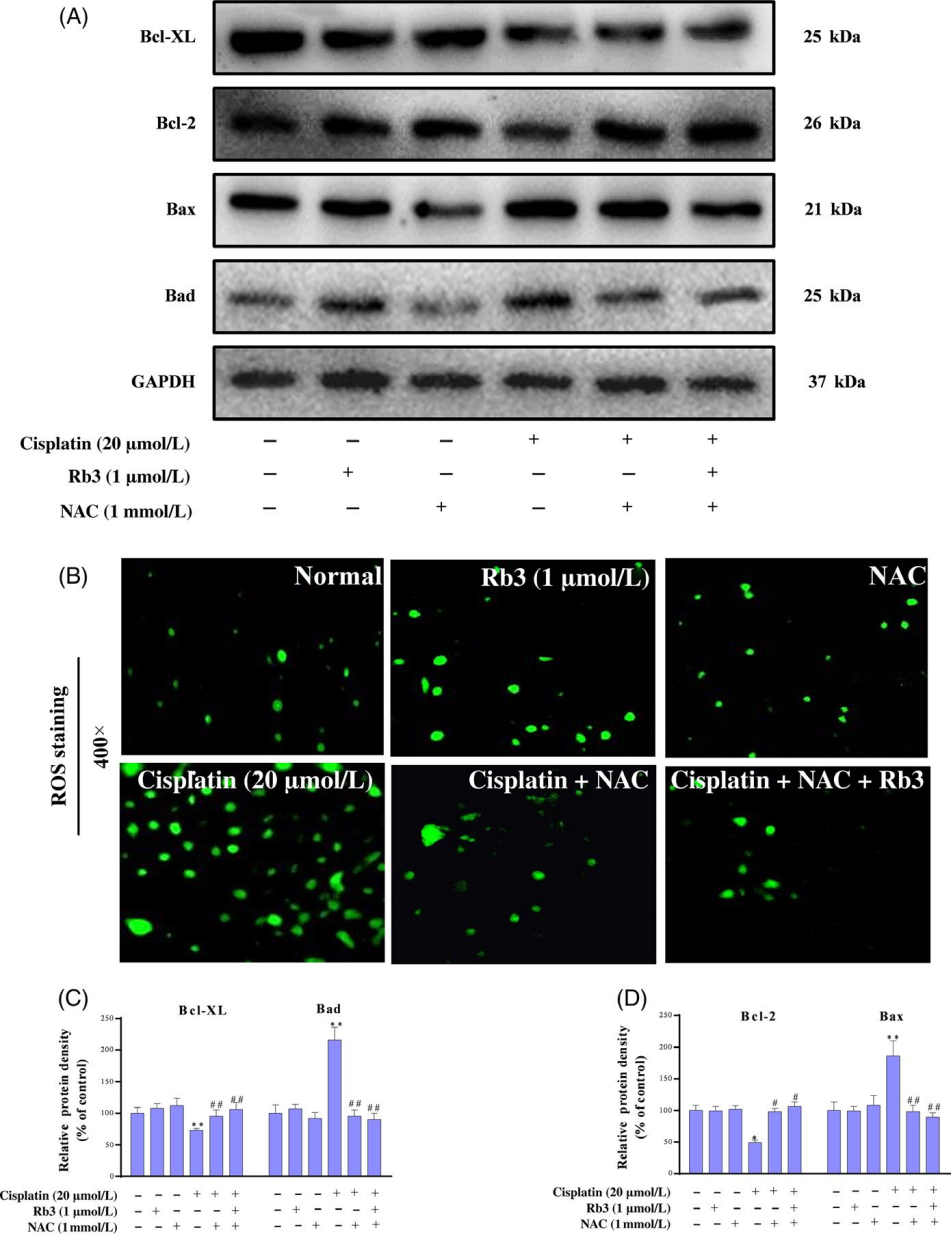
G‐Rb3 inhibits ROS‐mediated apoptosis evoked by cisplatin in cultured HEK293 cells. Effects of G‐Rb3 against cisplatin‐induced cytotoxicity by regulating apoptotic signal pathway. The protein expression levels of Bax, Bcl‐2, Bad and Bcl‐XL were measured by Western blot analysis with specific primary antibodies, and GAPDH protein level was used as a loading normal (A). The cell viability was determined by MTT assay. HEK293 cells were treated with cisplatin (20 µm), NAC (1 mmol/L), cisplatin plus NAC, and cisplatin plus NAC and G‐Rb3 (B). Effects of G‐Rb3 on cisplatin‐induced ROS generation. HEK293 cells (B) were stained with ROS staining. Quantification of Bcl‐XL, Bad, Bcl‐2 and Bax relative protein expressions was performed by densitometric analysis (C and D). All data are expressed as mean ± SD *P < 0.05 or **P < 0.01 comparing with normal group. ^#^ *P* < 0.05 or ^##^ *P* < 0.01 comparing with cisplatin group

Cisplatin‐induced apoptosis has been previously associated with both an intrinsic mitochondrial and an extrinsic death receptor pathway. To characterize the mechanism by which inhibition of ROS can increase the levels of apoptosis, we used an array of NAC, which have been associated with oxidative stress regulation. We identified cells treated with NAC with potential anti‐apoptosis functions whose expression was downregulated more than 1.5‐fold in HEK293 renal cells; re‐examination of these results with ROS staining showed that few green fluorescence was seen in NAC group; however, a large amount of green fluorescence was detected in cisplatin group. However, little was seen in the NAC plus cisplatin group. Interestingly, we found G‐Rb3 plus cisplatin and NAC cells showed the expected decrease in ROS production (Figure 6C). Therefore, the regulation of apoptosis may be possibly related to the generation of ROS. In order to elucidate the molecular mechanism underlying the reduction in apoptosis in response to G‐Rb3 treatment, we evaluated the expression level of Bcl‐2, Bcl‐XL, Bad and Bax by Western blot analysis (Figure 7A). The results showed that NAC could significantly reverse the decrease in Bcl‐2 and Bcl‐XL while downregulate the expressions of Bax and Bad. As we expected, G‐Rb3 plus cisplatin and NAC show a stronger change than NAC plus cisplatin cells. Therefore, these results showed that G‐Rb3 could exert inhibition of cisplatin‐evoked apoptosis in cultured HEK293 cells via downregulation of ROS generation.

### 3.7 Inactivation of AMPK signal pathway downregulates apoptosis and autophagy in cultured HEK293 cells

**Figure 8:**
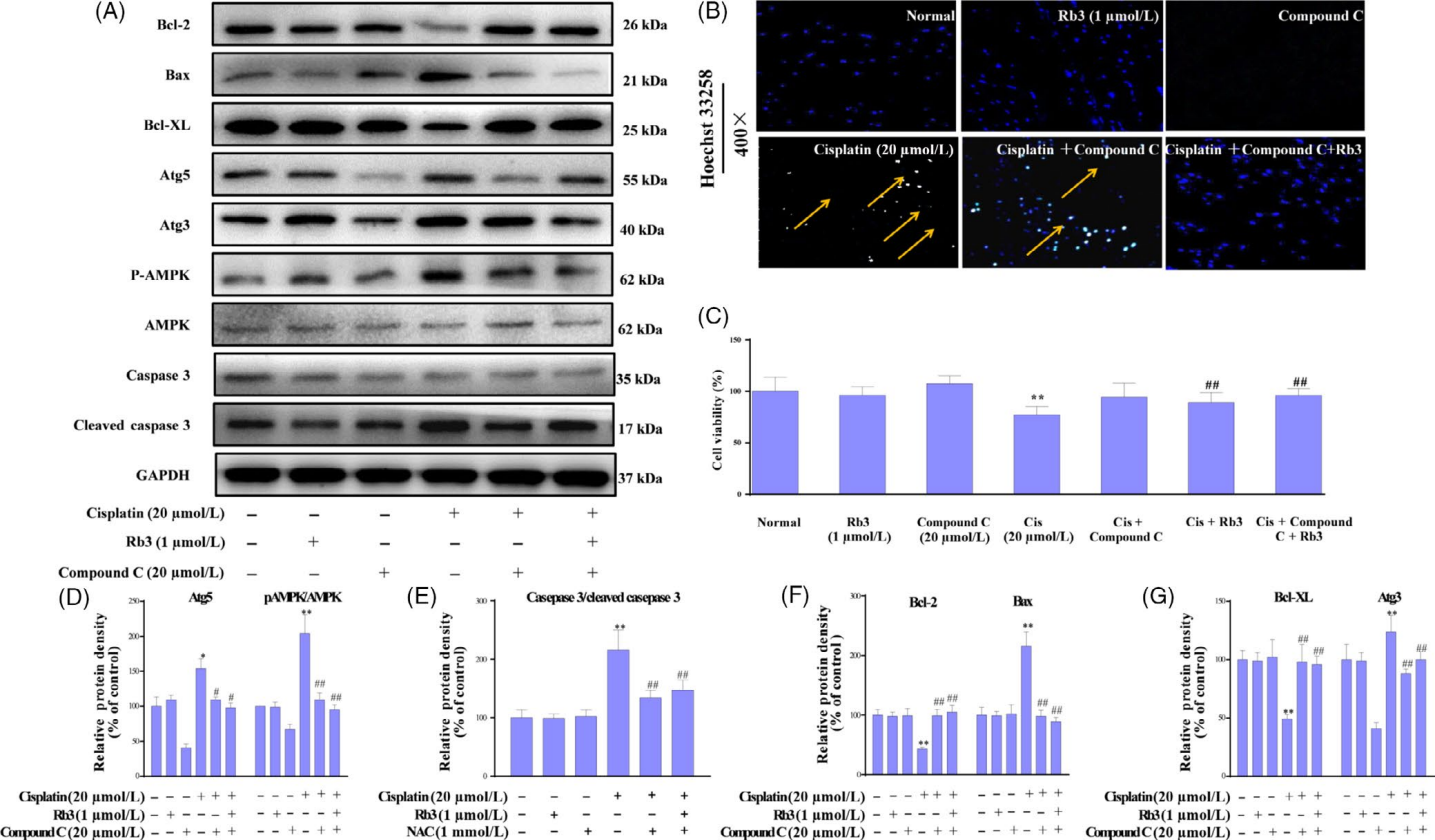
G‐Rb3 inhibits cisplatin‐induced apoptosis through AMPK signal pathway in cultured HEK293 cells. Effects of G‐Rb3 against cisplatin‐induced cytotoxicity by regulating apoptosis. The protein expressions of Bcl‐2, Bax, Bcl‐XL, Atg5, Atg3, AMPK and caspase 3 were measured by Western blot analysis with specific primary antibodies, and GAPDH protein level was used as a loading normal (A). Effects of G‐Rb3 on cisplatin‐induced apoptosis in HEK293 cells via Hoechst 33258 staining. For Hoechst 33258 staining, yellow arrows indicate apoptotic cells (B). The cell viability was assessed by MTT assay. HEK293 were treated with cisplatin (20 µm), compound C, cisplatin plus compound C, and cisplatin plus compound C and G‐Rb3 (C). Quantification of Atg5, AMPK, caspase 3, Bcl‐2, Bax, Bcl‐XL and Atg3 relative protein expressions was performed by densitometric analysis (D‐G). All data were expressed as mean ± SD *P < 0.05 or **P < 0.01 comparing with normal group. ^#^ *P* < 0.05 or ^##^ *P* < 0.01 comparing with cisplatin group

Previous reports have shown that cisplatin can lead to the activation of AMPK signal pathway, since their results may be susceptible to inactivation by AMPK. Thus, we investigated whether the changes of autophagy and apoptosis are due to the AMPK signal pathway. We incubated in vitro experiments with compound C of HEK293 cells, which had been treated or not with cells. We observed that the cells treated with compound C plus cisplatin were less efficient at AMPK, while much efficient at autophagy and apoptosis, suggesting that inactivation of AMPK was less active in cells where apoptosis was inhibited, probably due to their low levels of autophagy. We also confirmed that treatment with G‐Rb3 plus compound C and cisplatin shows remarkable decrease in apoptosis and autophagy. Hoechst 33258 staining further confirmed our results. Our results indicated that G‐Rb3 could exert inhibition of AMPK and then inhibit cisplatin‐induced apoptosis and autophagy (Figure 8C).

### 3.8 G‐Rb3 inhibits apoptosis via inhibition of autophagy in cultured HEK293 cells

**Figure 9:**
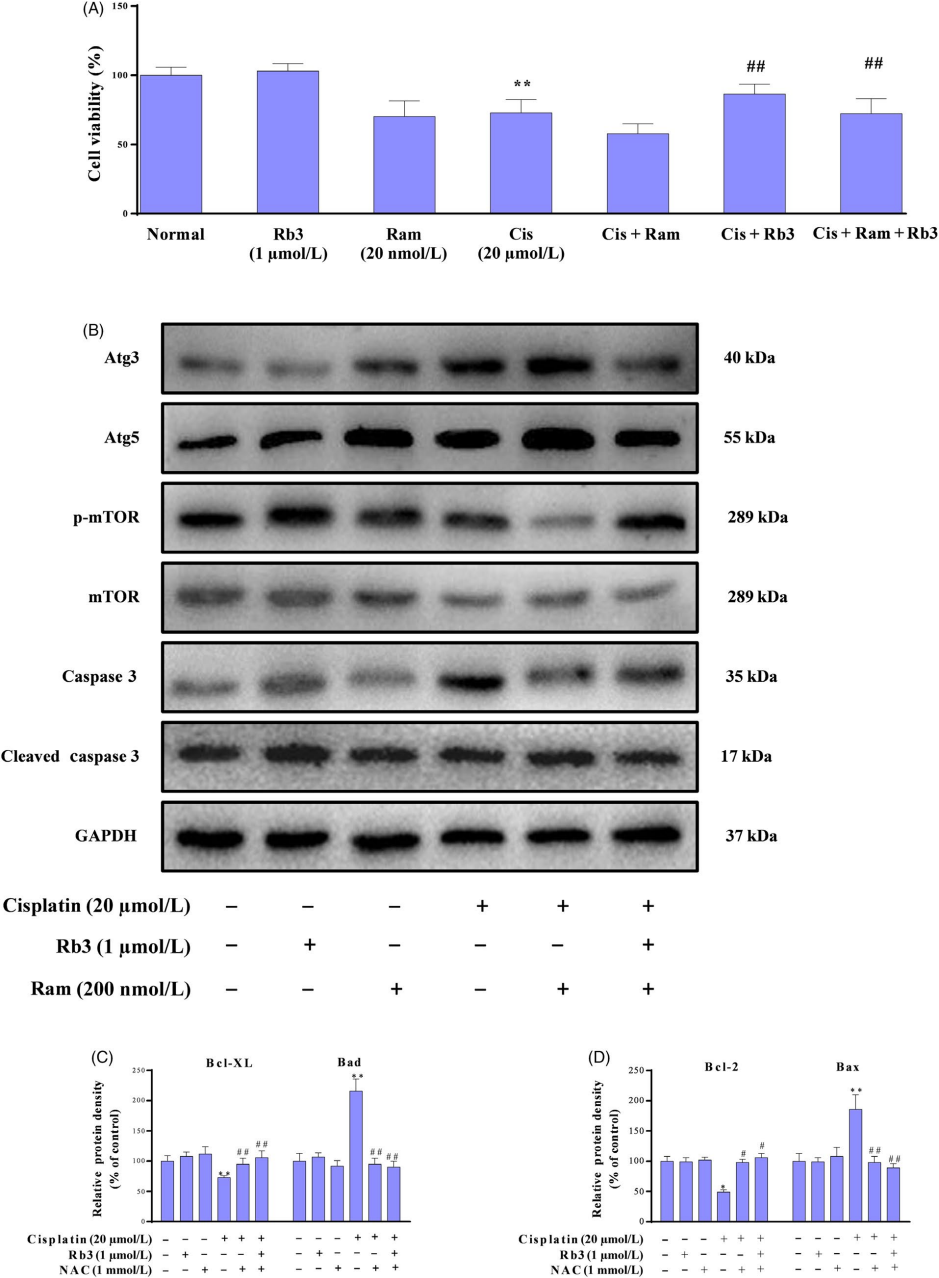
G‐Rb3 inhibits apoptosis via inhibition of autophagy in cultured HEK293 cells. Effects of G‐Rb3 against cisplatin‐induced cytotoxicity by regulating autophagy. The protein expressions of Atg3, Atg5, mTOR and caspase 3 were measured by Western blot analysis with specific primary antibodies, and GAPDH protein level was used as a loading normal (A). The cell viability was assessed by MTT assay. HEK293 cells were treated with cisplatin (20 µmol/L), Ram (200 nmol/L), cisplatin plus Ram, and cisplatin plus Ram and G‐Rb3 (1 µmol/L), respectively. Effects of G‐Rb3 on cisplatin‐induced apoptosis in HEK293 cells (B). Effects of G‐Rb3 on cisplatin‐induced apoptosis in HEK293 cells via Hoechst 33258 staining, yellow arrows indicate apoptotic cells (C). Quantification of Atg5, Atg3, caspase 3 and mTOR relative protein expressions was performed by densitometric analysis (D and E). All data were expressed as mean ± SD *P < 0.05 or **P < 0.01 comparing with normal group. ^#^ *P* < 0.05 or ^##^ *P* < 0.01 comparing with cisplatin group

To examine whether the activation of autophagy by rapamycin reproduced the apoptotic effect, the renal cells were exposed to cisplatin in the absence or presence of rapamycin and were subjected to Western blot experiments (Figure 9). As shown in Figure 9A, Western blot showed that rapamycin aggravated cisplatin‐induced autophagy and cell death. Interestingly, G‐Rb3 plus cisplatin and rapamycin attenuated cisplatin‐induced autophagy and apoptosis. Hoechst 33258 staining further confirmed our results.

## 4 DISCUSSION

Cisplatin is one of the most commonly used anti‐cancer agents.^25^ However, higher doses of cisplatin also induced severe kidney injury.^26^ Although specific mechanism of kidney injury caused by cisplatin is poorly understood, a growing body of evidence has illustrated that oxidative stress^27^, ^28^ and apoptosis^29^ play an important role in cisplatin‐induced AKI. Moreover, numerous studies have indicated that autophagy in the kidney tissue is rapidly induced by cisplatin exposure.^30^ Baek et al^22^ have confirmed that ginsenosides Rk3 and Rh4 (two rare ginsenosides isolated from Red ginseng) protect the kidney from cisplatin‐induced oxidative injury and help to recover renal function by restoring intrinsic antioxidant defences. However, its specific mechanisms have not confirmed. After comparing the viability of HEK293 cells pre‐treated with various ginsenosides under the treatment of cisplatin, we found G‐Rb3 could remarkably protect cells from damage caused by cisplatin exposure. Therefore, G‐Rb3 was tested in this report to evaluate its protective potential against cisplatin‐evoked kidney injury. The cellular events in cisplatin‐mediated nephrotoxicity, including decreased protein synthesis, membrane peroxidation, mitochondrial dysfunction and DNA injury, are a consequence of free radical generation and the body's inability to scavenge such molecules.^31^ Consistent with previous studies, we found that the renal dysfunction and morphological changes caused by cisplatin challenge were associated with oxidation and proximal tubular damage in mouse kidney. These findings were further confirmed by the results from in vitro HEK293 cells. Previous reports indicated that medicinal plants containing G‐Rb3 reduced oxidative stress and protected endothelial function in hypertension.^32^ Wang et al^33^ demonstrated G‐Rb3 extenuated oxidative stress. Xiao et al^34^ showed that G‐Rb3 protected kidney from ischaemia‐reperfusion damage through regulating the inhibition of apoptosis in rats.

In the current work, we demonstrated that G‐Rb3 pre‐treatment significantly attenuated cisplatin‐induced AKI through the decrease in apoptosis and autophagy. Cisplatin treatment caused typical symptoms and pathological changes in mice, including body weight loss, inflammatory infiltration and necrosis. In addition, cisplatin exposure induced damage to renal vasculature resulting in decline of glomerular filtration rate,^35^ which led to renal dysfunction, acute renal failure and elevation of serum BUN and CRE levels. G‐Rb3 significantly inhibited the elevation of BUN and CRE by cisplatin, suggesting positive effects of G‐Rb3 on renal dysfunction in cisplatin‐treated mice.

Previous studies have indicated that increasing formation of ROS, oxidative stress and lipid peroxidation contributes to renal histopathological changes after cisplatin challenge.^36^ Cumulative cisplatin leads to the production of large amounts of ROS in cells, leading to the development of oxidative stress.^37^ Previous reports have also clarified that oxidative stress is a central pathogenic factor in cisplatin‐induced AKI. Recently, an increasing number of studies confirmed that many natural products and active compounds exerted potential therapeutic values against cisplatin‐induced AKI.^12^, ^29^, ^38^ In these studies, G‐Rb3 administration for 10 consecutive days significantly alleviated AKI‐induced reduction in mice. G‐Rb3 decreased lipid peroxidation and restored antioxidant capacity of kidney manifested by an elevation of SOD activity and GSH content as well as a reduction in MDA content. Therefore, this report reconfirmed that ROS was regulated by G‐Rb3, which supported the evidence provided by Monica et al.^35^

Cisplatin induces apoptosis mainly through the mitochondrial (intrinsic) signal pathways.^28^ Tubular cell apoptosis is a characteristic feature of cisplatin nephrotoxicity, which results in the loss of renal endothelial cells and renal dysfunction.^29^ The induction of apoptosis through the intrinsic signal pathway is regulated primarily by Bcl‐2 family proteins, notably the pro‐apoptotic Bax and anti‐apoptotic Bcl‐2 proteins. The release of cytochrome c from mitochondria and subsequent cell death is prevented when Bax forms heterodimers with Bcl‐2.^28^ In this report, cisplatin induced apoptosis with downregulation of Bcl‐2 and upregulation of Bax. Concomitantly, the number of TUNEL‐positive cells increased significantly after the cisplatin injection. G‐Rb3 resulted in a significant reduction of positive cells for TUNEL staining. Evidently, apoptosis in AKI is regulated by the balance of anti‐ and pro‐apoptotic proteins in the Bcl‐2 and caspase family,^39^ and activation of caspase 3 and caspase 8 is critical execution factors of caspase‐dependent apoptosis.^40^ It was also shown in this study that single exposure of cisplatin dramatically increased the protein levels of Bax, cleaved caspase 3 and cleaved caspase 8, while decreased Bcl‐2. Pre‐treatment of G‐Rb3 restored the balance of anti‐ and pro‐apoptotic proteins in kidney as compared with that in the cisplatin‐injured group mice.

In addition, G‐Rb3 pre‐treatment significantly attenuated the increase in ROS levels, our results were confirmed by NAC through ROS staining in HEK293 kidney cells. There is growing evidence showing that ROS‐mediated apoptosis is one of the most important pathological mechanisms of cisplatin‐caused nephrotoxicity. When cisplatin accumulates in the mitochondrial matrix, it causes a large amount of ROS production and mitochondrial dysfunction, leading to increased mitochondrial permeability, pro‐apoptotic factor release and initiate apoptosis. The Bcl‐2 family of proteins has a crucial role in the apoptotic mechanism of mitochondrial pathway, which includes pro‐apoptotic protein Bax and anti‐apoptotic protein Bcl‐2.^41^ Bax is capable of activating caspase 9, which is responsible for proteolytic activation of caspase 3 involved in the cleavage of a group of proteins. In contrast, Bcl‐2 exerts its anti‐apoptotic activity by inhibiting the translocation of Bax to mitochondria.^42^ In this study, we observed increases in Bax, Bad and cleaved caspase 3 and decrease in Bcl‐2 and Bcl‐XL expression levels in cisplatin group. NAC decreased the apoptosis induced by cisplatin, while NAC plus cisplatin and G‐Rb3 showed remarked decrease in the cellular apoptosis confirmed by Western blot in comparison with cisplatin plus NAC group in HEK293 renal cells.

ROS are known to trigger autophagy in mammalian cells and tissues.^43^ Mitochondria abundantly distributed in the renal cells are vulnerable to the ROS‐triggered mitochondrial dysfunction with loss of mitochondrial membrane potential along mitophagy.^44^ Autophagy plays a protective role in a number of clinically important disorders. In NRK‐52E cells, autophagy provided protection from a lower dose (10 µmol/L) cisplatin injury. Also in NRK‐52E cells, suppression of autophagy either by autophagy inhibitors or by beclin‐1 siRNA prevented apoptosis.^45^ We have addressed that G‐Rb3 could reverse apoptosis induced by cisplatin in a mouse model and in HEK293 cells. In this regard, we hypothesized that autophagy may play a vital role in cisplatin‐induced nephrotoxicity.

There is little knowledge to date on the relationship between autophagy and cisplatin treatment and on the mechanisms of cisplatin‐induced autophagic response to nephrotoxicity.^46^ MTOR serves as a central regulator by negatively regulating autophagy. AMPK inhibits mTOR and initiates autophagy.^47^ Thus, the AMPK/mTOR signalling pathway may be involved in regulating autophagy in response to various anti‐cancer agents.^48^ The formation of autophagosome through expression of autophagy‐related genes and the conversion of LC3‐I to LC3‐II through photolytic cleavage and lipidation are considered hallmarks of mammalian autophagy.^49^ Glucose deprivation contributes to ATP decrease and ROS accumulation, which in turn activates AMPK.^50^ AMPK, a cell energy sensor, is activated in response to low glucose oxidative stress and activates metabolic reprogramming under certain environmental conditions during autophagy.^51^ The mechanism of AMPK‐induced autophagy presumably involves downregulating of mTOR, which is known as an important central negative regulator of autophagy.^52^, ^53^ Previous studies have reported that activation of AMPK induces an autophagy response in tumour cells.^54^ AMPK is a key regulator of autophagy in many cell types.^16^ In current experiments, G‐Rb3 suppressed cisplatin‐evoked autophagy in mice. G‐Rb3 increased the level of p‐AMPK in HEK239 cells. G‐Rb3 has been shown to alter the steady‐state level or ratio of LC3‐II:LC3‐I, indicating a significant autophagosome formation.^55^ In the current study, cisplatin exposure increased the protein expression levels of Atg3, Atg7 and Atg5, which were in line with the increase in the ratio of LC3‐II/LC3‐I, indicating an activation of autophagy. Interestingly, pre‐treatment with G‐Rb3 (10 and 20 mg/kg) significantly decreased the protein expressions of these proteins in renal tissues. BNIP3,^56^ a mitochondrial pro‐apoptotic protein belonging to the Bcl‐2 family, is activated by the cisplatin‐induced damage of renal tubular epithelial cells and is considered as an adaptive response to pathogenetic oxidation in AKI.^57^, ^58^ Additionally, LC3‐I was accumulated in Cisplatin plus Rb3 groups ,and higher than that in cisplatin‐treated alone mouse kidney (shown in Figure 4A). The illustrated data in this report demonstrated that the changes in AMPK signalling were partially counteracted in the presence of G‐Rb3, further indicating that the ROS could at least be partially involved in signalling pathways through activation of AMPK. Autophagy and apoptosis are highly interconnected, but this relationship has not been well elucidated.^59^ Thus, the pivotal molecular mechanism behind the regulation of autophagy and apoptosis induced by cisplatin should be further explored. Here, we deployed an autophagy inhibitor to reassure the role of autophagy, whether autophagy plays a negative role in nephrotoxicity, and if yes, in which way it participated. In agreement with these observations, the results from Western blot analysis confirmed that Ram remarkably increased distribution of autophagy‐related proteins such as Atg3 and Atg5 in HEK293 renal cells during cisplatin treatment. Interestingly, G‐Rb3 inhibited the increase in these proteins, indicating that G‐Rb3 exerted restraint effects to autophagy. Moreover, Hoechst 33258 staining assay provided independent evidence that cisplatin and Ram treatment triggered apoptosis, while G‐Rb3 significantly enhanced the proliferative effect on HEK293 renal cells compared to cisplatin alone. Our present study allows us to conclude that autophagy appeared to have a negative role in protecting HEK293 cells from a cisplatin‐mediated apoptosis effect.

In this study, we elucidated that autophagy triggered by cisplatin was mediated by activation of the AMPK/mTOR signal pathway in mice and HEK293 cells. Treatment of mice and cells with cisplatin augmented protein expression levels of phosphorylated AMPK, downregulated phosphorylated mTOR protein and upregulated LC3‐II and Atgs. In agreement with these findings, the AMPK inhibitor compound C plus cisplatin suppressed phosphorylation of AMPK and increased Atgs levels, as compared with the cisplatin alone treatment. Collectively, autophagy is a key pathway of cisplatin‐induced nephrotoxicity, and the suppression of autophagy *via* AMPK/mTOR by G‐Rb3 may antagonize the damaging effect through reduced accumulation of ROS.

**Figure 10:**
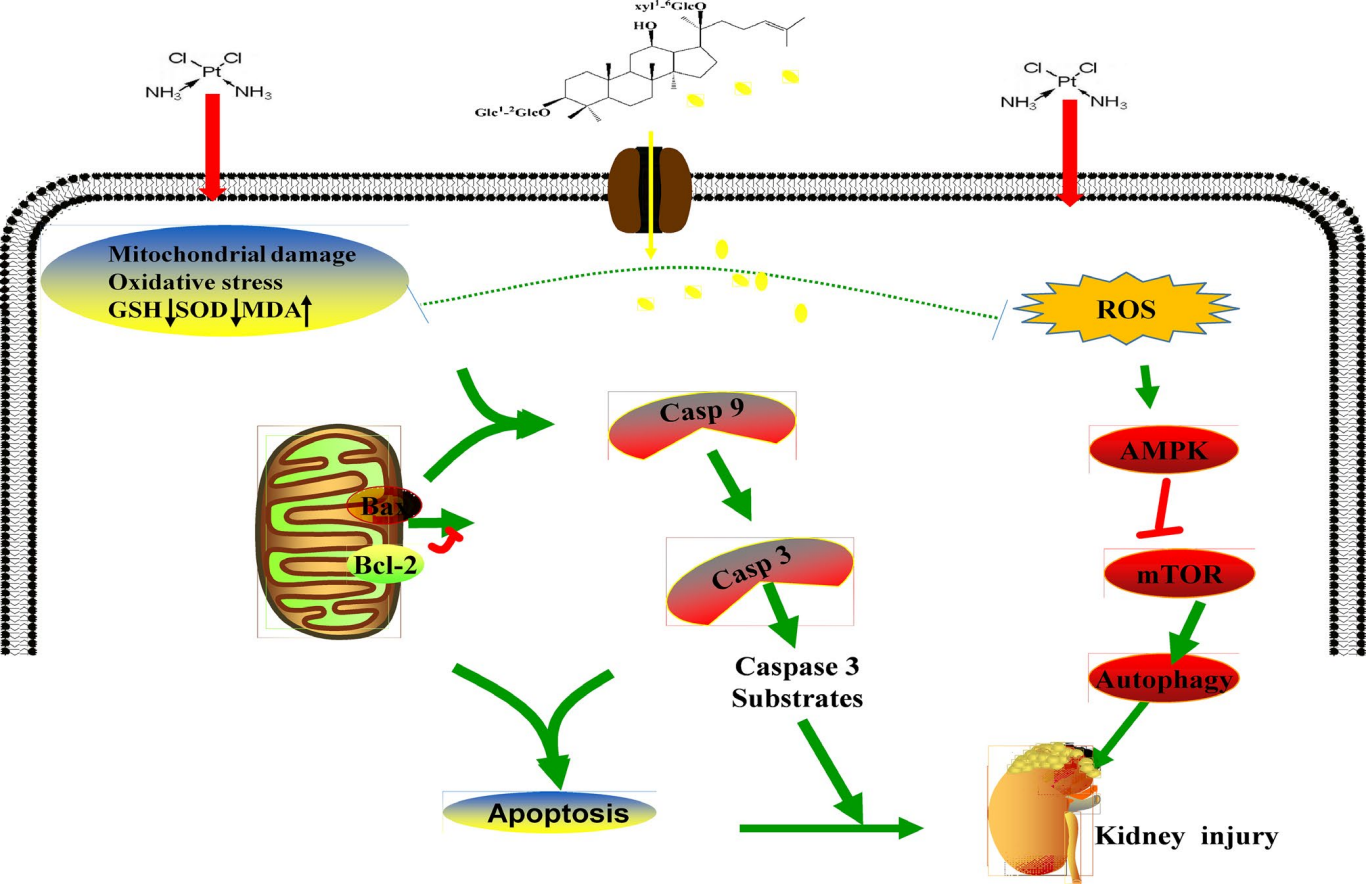
Scheme summarizing the inhibition of cisplatin‐induced nephrotoxicity by G‐Rb3 via the downregulation of AMPK‐/mTOR‐mediated autophagy and inhibition of apoptosis. The release of ROS can be promoted by cisplatin, which can activate the AMPK signal pathway and then activate autophagy, resulting in kidney injuries. However, G‐Rb3 can reduce autophagy by reducing oxidation and apoptosis, thereby alleviating cisplatin‐evoked kidney damages

As summarized in Figure 10, this research demonstrated, for the first time, the protective effects and the underlying mechanism of G‐Rb3 against cisplatin‐induced renal failure, through regulating AMPK/ mTOR signal pathway to restore antioxidant systems and to inhibit proximal tubular injury by suppression of ROS‐mediated apoptosis and autophagy. Specifically, G‐Rb3 treatment reversed the levels of MDA, SOD and GSH. Furthermore, G‐Rb3 inhibited the activation of Bax, Bad, caspase 3 and caspase 9 and increased Bcl‐2, to reduce tubular cells damage, nuclear fragmentation and condensation.

In conclusion, we have demonstrated that G‐Rb3 alleviates cisplatin‐caused cytotoxicity in renal tubular cells and restores kidney function in a mice model. Ginsenoside Rb3 could be viable therapeutic agents or adjuvants that reduce nephrotoxicity during cisplatin chemotherapy.

## CONFLICTS OF INTEREST

The authors declare no conflict of interest.
